# The Effect of Cumulative Lifetime Estrogen Exposure on Cognition in Depressed Versus Non-Depressed Older Women

**DOI:** 10.1177/08919887221090216

**Published:** 2022-04-11

**Authors:** Hanadi Ajam Oughli, Sarah A. Nguyen, Prabha Siddarth, Molly Fox, Michaela Milillo, Linda Ercoli, Helen Lavretsky

**Affiliations:** 1 Department of Psychiatry, Semel Institute for Neuroscience, 8783 University of California Los Angeles, Los Angeles, CA, USA; 2 Department of Anthropology, 8783 University of California Los Angeles, Los Angeles, CA, USA

**Keywords:** depression, lifetime estrogen exposure, cognitive function, women’s health, reproductive life history

## Abstract

**Objectives:**

Two-thirds of individuals living with Alzheimer’s disease are women. Declining estrogen levels influence mood and cognition. Cumulative lifetime estrogen exposure (CLEE) correlates with cognition later in life. We examined the relationship of CLEE to depression and cognition in older women with major depression compared to non-depressed women.

**Design:**

Older women (age ≥60 years) with depression were compared to non-depressed women using a lifetime estrogen exposure questionnaire. CLEE was defined as combined durations of reproductive span (age of menopause minus age of menarche) and any post-menopausal hormone replacement therapy use. Higher vs lower CLEE groups were based on a median of 474 months of estrogen exposure.

**Setting:**

University hospital outpatient research program

**Participants:**

135 women ≥60 years; 64 depressed and 71 non-depressed

**Measurments:**

Participants completed a comprehensive cognitive test battery. General linear models were used to examine the association between cognitive domain scores and CLEE in depressed and non-depressed women, controlling for age, education, and ethnicity.

**Results:**

Depressed and non-depressed groups had significantly different levels of CLEE, measured in months: mean 495.7 (SD 108.6) vs 456.4 (SD 66.0) months, F(1,130) = 5.01, p = .03. Within the non-depressed participants, higher CLEE was associated with improved delayed recall (F(1,59) = 5.94, p = .02, effect size = .61), while no such relationship was observed in the depressed group.

**Conclusion:**

Higher CLEE was associated with improvement in delayed recall among non-depressed, but not among depressed participants. This suggests a protective role of estrogen on memory in non-depressed older postmenopausal women. Further research should examine the role of the CLEE in antidepressant response and cognitive decline.

## Introduction

Women develop cognitive and mood disorders at a higher rate than men across the lifespan.^1,2^ While some studies have not found sex differences in the incidence of Alzheimer’s disease (AD),^3^ more recent studies have shown that the risk of AD is two to three times higher in women than men,^4,5^ with women exhibiting more significant cognitive impairment at the same AD stage.^6^ Some of the sex differences in AD prevalence can be attributed to differences in longevity, but there also appear to be distinct biological mechanisms that increase the progression of AD in women, such as deviations in brain structure and biomarkers, the impact of pregnancy, menopause, sex hormones, genetic backgrounds, and inflammation.^4,7^ The etiology and the sex-specific risk factors underlying these vulnerabilities remain insufficiently understood, and as a result, hormone-influenced sex differences in psychopathology have been extensively studied.^8,9^

Estrogen, synthesized in the ovaries and the brain, is involved in cognition, memory, learning, and mood.^9^ This has been demonstrated by in vitro and animal systems research which have shown that estrogen modulates brain networks and processes implicated in AD (e.g., inhibiting amyloid-β formation,^10^ neuronal apoptosis,^11^ and tau hyperphosphorylation^12)^ as well as major depressive disorder (e.g., stress response and emotion regulation).^13^ However, the role of cumulative estrogen exposure on cognition remains elusive. A few studies have found no association between estrogen exposure and dementia risk^14,15^ or cognitive performance.^15,16^ Findings from the Rotterdam cohort showed that women with a longer reproductive span had an increased risk of dementia, with the risk being more pronounced among (APOEε4) carriers.^17^ In contrast, a range of studies have shown that cumulative lifetime estrogen exposure (CLEE), defined as the sum of endogenous exposure throughout women’s reproductive period and exogenous exposure to hormone replacement therapy,^18^ was found to be correlated with improved memory, and particularly with prospective memory performance in later life, thereby improving independent everyday function and quality of life.^18,19^ Additionally, the added duration of reproductive span and hormone therapy use was associated with less cognitive decline and AD risk and an overall improvement in global cognitive functioning.^20,21^

Late-life depression (LLD) is also associated with a higher risk for AD, cognitive dysfunction, and with a poor response to antidepressants.^22^ Older adults with depression tend to perform worse on executive function, memory, and processing speed measures.^22^ The degree of cognitive impairment in LLD may further correlate with the risk for developing dementia.^23^ While mild cognitive impairment associated with LLD often does not progress to dementia, severe cognitive dysfunction in LLD is associated with a higher risk of developing dementia.^23^ The heterogeneity in cognitive abnormalities found in LLD plays a key role in the prognosis, severity, and course of the disease, as well as treatment outcomes.^23^ Furthermore, there are no known studies to date examining the potential neuroprotective role of estrogen on cognition in older women with LLD. The purpose of this study is to examine the relationship between CLEE and cognition in older women with and without depression. We hypothesized that higher CLEE would be associated with better mood and cognitive function in older women with or without depression.

## Methods

We present a cross-sectional analysis of 2 convenience samples collected from 2 randomized controlled trials with similar assessment protocols. Baseline measures were used in this analysis, prior to the implementation of study interventions.

Depressed participants aged 60 years or older were recruited from the “Brain Connectivity and Response to Tai Chi Geriatric Depression” study (NCT02460666), conducted at the University of California Los Angeles (UCLA), where they were randomized to either a 12-week Tai Chi Chih (TCC) intervention or 12-week Health and Wellness Education intervention. Participants were included if they had met criteria for an episode of major depression, as assessed by a Hamilton Rating Scale for Depression (HAMD) score >14 at baseline, despite being on stable antidepressant treatment for at least 4 months. Additionally, participants required a Mini-Mental Status Exam (MMSE) score >24 at baseline.

Exclusion criteria included other psychiatric disorders including psychosis, bipolar and substance use disorders, and dementia as ascertained by the Structured Clinical Interview DSM-5 (SCID) (except for frequently comorbid anxiety or insomnia). The diagnosis of dementia was further evaluated through an extensive history review, MMSE score of <24, and a clinical dementia rating score of CDR >.5. We also used performances between −1.5 and 2 (SD) below age and education norms on one of two memory tests from our cognitive battery to establish cutoffs according to a widely accepted practice for the diagnosis of dementia.

Additionally, participants were excluded if they responded to effective antidepressant treatment or psychotherapy as determined by an extensive review of psychiatric history, have unstable medical or neurological disorders such as myocardial infarction within 6 months or surgery within the last 3 months, or any disabilities due to mobility problems that precluded them from participating in the TCC exercise intervention.

Participants received an initial medical evaluation including a complete physical examination with neurological examinations, electrocardiogram (ECG), and laboratory testing at baseline to rule out medical illnesses that could account for behavioral and cognitive symptoms. Furthermore, all participants were interviewed about their history of psychiatric and medical illnesses, psychosocial stressors, current medications, and health status.

Participants were also excluded if they had prior experience with practicing a mind–body intervention (defined as Tai Chi, Tai Chi Chih, yoga, and meditation) or have practiced it at least weekly within the past 12 months in order to minimize achieving a ceiling effect of these interventions in those who have learned the techniques and are actively practicing.

Neuropsychological assessments were administered in-person at baseline, week 12, and at 6, and 12 months. The baseline assessments were performed within 2 months prior to initiation of the intervention.

Non-depressed participants were recruited from the “Reducing Risk for Alzheimer’s disease in High Risk Women through Yoga or Memory training” study (NCT03503669), conducted at UCLA, where they were randomized to either a 12-week trial of memory enhancement training (MET) intervention or a combination of Kundalini Yoga (KY) and Kirtan Kriya (KK) yogic meditation. Inclusion criteria included current subjective memory complaints, MMSE score >24, high cardiovascular risk based on the 2013 ACC/AHA Guideline on the Assessment of Cardiovascular Risk, sufficient English proficiency, and capacity to provide informed consent. Additionally, participants needed to be naïve to MET and KY+KK and have not practiced in the past year as this could lead to confounding effects.

Exclusion criteria included a diagnosis of dementia as diagnosed by DSM-5 criteria, an MMSE of ≤23, any psychiatric diagnoses including bipolar disorder, psychosis, alcohol/drug dependence or neurological disorder, clinically significant depressive symptoms as indicated by a Beck Depression Inventory (BDI) score >17 during the baseline screening visit, unstable medical conditions such as myocardial infarction within the past 6 months or surgery (within 3 months) or anticipated surgery within the next year, any disability preventing participants from engaging in MET or KY such as severe visual or hearing impairment, current yoga practice, currently taking psychoactive medications, or having received or currently participating in cognitive training for memory or other cognitive symptoms.

All participants underwent baseline physical examinations as well as laboratory tests. Neuropsychological assessments were administered in-person at baseline, week 12, and at 6, and 12 months or upon early termination.

Neuropsychological assessments were administered by trained raters who were blind to the intervention assignment. All study procedures were approved by the UCLA Institutional Review Board.

### Estrogen Exposure

All participants were administered a lifetime estrogen exposure questionnaire via phone or email at any point throughout the study (see Supplemental material). The items in the questionnaire were written by consensus among the research team adapted from previous relevant work^19^ guided by the anthropological concept of “reproductive life-history,” that is, the timing and implementation of reproductive events across the life cycle. Several studies in this area have taken the approach of using reproductive span (years between menarche and menopause) or estrogen span (reproductive span + ERT) to proxy cumulative lifetime estrogen exposure.^19^

We highlight the importance of distinguishing between the dose (quantity) of estrogen exposure and the duration of estrogen exposure.^24^ While exogenous hormone therapy such as oral contraceptives and pregnancy alters the quantity of estrogen to which a women is exposed to each month of her life, they do not alter the duration of her estrogen exposure of the alternative state is ovulatory menstrual cycles.^24^ As for the distinction between exogenous estrogen, including oral contraceptives and HRT, we note that exogenous estrogen therapies are composed of conjugated equine estrogen (CEE), which contains estrone and 17β-estradiol in addition to other estrogens.^25^ We have no a priori reason to suspect that CEE should exert different biological effects than the bioidentical endogenous equivalents.

Our questionnaire aimed to capture the duration of estrogen exposure through detailed elements of the reproductive life-history that are and are not typically available in medical records. Thus, in this study, endogenous estrogen exposure was calculated as age in months at menopause minus age at menarche.^21^ Exogenous estrogen exposure was defined as any post-menopausal hormone replacement therapy use.^21^ Cumulative lifetime estrogen exposure (CLEE) was calculated by combining endogenous and exogenous estrogen exposures. Participants were then divided into higher vs lower CLEE groups based on a median of 474 months of estrogen exposure.

### Cognitive Measures

Both clinical trials used a similar cognitive assessment schedule. Participants completed a comprehensive neuropsychological test battery that evaluated the following cognitive domains: learning (California Verbal Learning Test-II [Trials 1 through 5 Total] (Tai Chi study) or Hopkins Verbal Learning Test-Revised [Total Recall, Trials 1 - 3] (KY study), Rey–Osterrieth Complex Figure Test [3-minute recall]); delayed recall (California Verbal Learning Test-II [long delayed free recall] or Hopkins Verbal Learning Test [Delayed Recall], Rey–Osterrieth Complex Figure Test [30-minute delayed recall]); and executive functioning (Trail Making Test B, Controlled Oral Word Association test [FAS]).

The California Verbal Learning Test-II (CVLT-II) is a multi-trial verbal memory test that involves learning 16 words over 5 trials, followed by short-delay and long-delay cued and free recall and recognition testing. The words can organized into 4 semantic categories. A distraction list is also presented. Clinically, the CVLT-II has been used to distinguish among patients with dementia, mild cognitive impairment, and normal aging.^26^ The CVLT-II has 2 equivalent forms.

The Hopkins Verbal Learning Test-Revised is a multi-trial verbal memory test that consists of three-trial learning for 12 words, followed by immediate and delayed free recall and recognition testing. The words can be grouped into 3 semantic categories.^27^ It has 6 equivalent alternate forms and is appropriate for serial testing as part of longitudinal studies.^27^ Clinically, the HVLT-R has been used to distinguish mild cognitive impairment from normal aging.

The CVLT-II and HVLT-R share several important properties; both use multiple learning trials to ensure learning with immediate and delayed recall and recognition testing formats. Both include words that can be grouped by semantic categories. Furthermore, both of these tests measure the construct of verbal learning, and the scores used in the current study (the Total Recall and Delayed Recall scores) are significantly correlated.^28,29^

The Rey–Osterrieth Complex Figure Test (ROCF) is a widely used neuropsychological test for the evaluation of visuospatial constructional ability and visual memory.^30^ It consists of 3 test conditions; copy, immediate recall, and delayed recall.^30^

The Trail Making Test (TMT) is an indicator of executive function, graphomotor speed, and visual scanning (Parts A and B), and cognitive set-shifting (Part B only).^31^ TMT is a valid test and is used to distinguish neurological patients from healthy elders and is used as an indicator of cognitive dysfunction.^31^

Controlled Oral Word Association test (FAS) is a sensitive and brief measure of verbal fluency and requires takers to name as many words, starting with a single letter, as they can in 1 minute. It is also considered to be a test of executive functions and tends to be sensitive to cognitive dysfunction disorders that affect executive function.^32^

### Statistical Analysis

Prior to analyses, data were inspected for outliers, skewness, and homogeneity of variance to ensure the appropriateness of parametric statistical tests. T-tests and chi-square tests were used to examine demographic differences between depressed and non-depressed participants. For the neuropsychological measures, raw scores were transformed to *z*-scores for each test score for each participant based on sample means. *Z-*scores were reversed when necessary so that high *z*-scores represented good performance for all measures. These *z*-scores were averaged within each neuropsychological domain to produce composite scores. General linear models were used to examine whether cognitive domain scores were associated with CLEE in depressed and non-depressed women, controlling for age, educational level, body mass index (BMI), and ethnicity. Since depression is known to be associated with worse cognitive performance,^22^ we included depression status as a covariate and also examined the interaction term of CLEE and depression status. Follow-up analyses (investigating each group separately) were conducted for those domains with significant interaction. All available baseline data from the 2 trials were used in the analyses. Statistical analysis was performed using SAS version 9.4 (SAS System for Windows, Cary, NC), and P < .05 was regarded as statistically significant.

## Results

A total of 135 participants aged 60 years and older were included in the analysis. Sixty-four women with depression, of which 3 had a diagnosis of mild cognitive impairment (MCI), (mean age 69.1 (SD 6.4, range 60–86) years) were compared to 71 non-depressed women (mean age 66.1 (SD 8.3, range 55–86) years), of which 2 had a diagnosis of MCI. Women with depression had a mean HAMD score of 18.83 (SD 3.63), indicative of moderate depression.^33^ Non-depressed women had a mean BDI score of 7.41 (SD 4.92) indicative of mild depressive symptoms endorsed by some participants.

Table 1 compares the groups in terms of baseline characteristics and cognitive measures. There were no group differences by race, education, BMI, MMSE, and other cognitive measures. However, depressed participants were significantly older than the non-depressed women.

**Table 1.** Baseline demographic characteristics and cognitive measures of depressed and non-depressed participants.

	Non-Depressed Participants	Depressed Participants	Chi Square/t Statistics (DF)	*P*-value
	(n = 71)	(n = 64)		
Demographics				
Age	66.1 (8.3)	69.1 (6.4)	2.4 (133)	.02
Education	16.0 (1.9)	15.9 (1.8)	.3 (133)	0.8
Race (%)				0.2
Caucasian	45 (63.4%)	53 (82.8%)		
African American	10 (14.1%)	4 (6.3%)		
Asian	6 (8.5%)	4 (6.3%)		
Other	5 (7.0%)	2 (3.1%)		
Hispanic	5 (7.0%)	1 (1.6%)		
Body mass index	27.4 (6.3)	26.0 (5.7)	1.3 (124)	0.2
Cognitive measures				
MMSE	28.4 (1.4)	28.8 (1.2)	1.7 (133)	.09
Learning^a^	.09(1.0)	.09(1.0)	1 (126)	0.3
Delayed recall^a^	−.05 (.9)	.05 (1.0)	.6 (126)	0.5
Executive functioning^a^	.03 (1.2)	−.04 (.8)	.4 (126)	0.7
Processing speed^a^	−.11 (.9)	.12 (1.0)	1.3 (126)	0.2
Depression				
HAM-D^b^	NA	18.8 (3.6)		
BDI^c^	7.41(4.92)	NA		
Estrogen exposure measures				
CLEE^d^	456.9 (64.9)	494.3 (109.0)	2.4 (133)	.02
CLEE group^d^			2.1 (1)	0.1
Lower CLEE group	40 (56.3%)	28 (43.7%)		
Higher CLEE group	31 (43.7%)	36 (56.3%)		

Abbreviations: MMSE, Mini-Mental Status Exam; CLEE, Cumulative Lifetime Estrogen Exposure; DF, Degrees of Freedom.

^a^Z-scores.

^b^Hamilton Rating Scale for Depression, 24-item.

^c^Beck Depression Inventory.

^d^Participants were divided into higher vs lower CLEE groups based on a median of 474 months of estrogen exposure.

Depressed participants had significantly higher levels of CLEE compared to non-depressed participants: 495.7 (SD 108.6) vs 456.4 (SD 66.0) months, F(1,130) = 5.01, p = .03, controlling for age, BMI, and ethnicity. The association between depression status and CLEE exposure groups was not statistically significant, χ^2^ (1) = 2.13, p = .1. In other words, the effect was significant when CLEE was considered as a continuous variable but did not reach significance when categorical.

**Figure 1.**
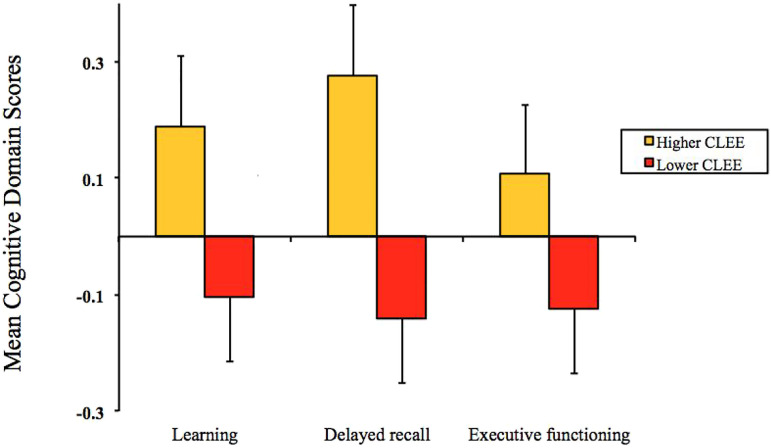
Learning, delayed recall, and executive functioning by CLEE groups in non-depressed participants. Error bars represent standard deviation.

Controlling for age, educational level, BMI, ethnicity, and depression status, CLEE was not associated with any of the cognitive domain scores in the combined sample of depressed and non-depressed participants. The interaction term of depression status and CLEE was associated only with delayed recall: F (1,127) = 3.79, p = .05. Follow-up analyses conducted within each group separately revealed that within the non-depressed group, CLEE was significantly associated with performance on delayed recall: the higher CLEE group performed better in delayed recall than the lower CLEE exposure group (.23 (.77) vs −.28 (.89), F (1,59) = 5.94, p=.02, effect size = .61), see Figure 1. Within the depressed group, CLEE was not significantly associated with delayed recall performance (F (1,56) = .08, p = .8.

## Discussion

This is the first study to examine CLEE on cognitive function in depressed and non-depressed older women. We found that higher CLEE was associated with better performance on delayed recall in women without depression; however, learning and executive function did not differ by lower vs higher CLEE groups. In addition, CLEE was not associated with cognitive function in the depressed group. Our findings are supportive of the estrogen exposure influencing cognitive performance in non-depressed but not in depressed older women.

Our findings are consistent with previous studies which showed that earlier age of menarche, later age at menopause, and increased duration of estrogen therapy were associated with lesser cognitive decline,^21^ a reduced risk of Alzheimer’s disease, and dementia.^34^ Longer reproductive periods were associated with better immediate and delayed verbal memory and working memory, while previous hormone therapy usage was associated with better verbal memory and delayed visual memory.^7^ Estrogen therapy was also found to enhance working memory and frontal mediated tasks.^35,36^ Furthermore, higher CLEE was noted to improve prospective memory performance later in life.^18^

The precise mechanism by which CLEE may influence cognitive performance in later life remains unclear. One explanation might be that estrogen has neuroprotective effects on memory, thus playing a significant role in brain aging.^20^ Estrogen receptors have been found to be expressed in the human dorsolateral prefrontal cortex and hippocampus,^37,38^ and estrogen itself has been found to be capable of modulating regional cerebral blood flow and brain activation patterns in the dorsolateral prefrontal cortex in women.^39,40^ Therefore, beneficial structural and metabolic changes could result from longer estrogen exposure, ultimately leading to better cognition and memory in older non-depressed women.

We did not find significant associations between CLEE and cognitive performance in the depressed group. This is in contrast to previous studies that showed that estrogen replacement therapy was associated with greater global improvement in cognitive functioning in older depressed women and appeared to augment antidepressant response to SSRIs.^41^ One explanation could be that the severity of the depressive symptoms in our sample could have overpowered any benefit from increased estrogen exposure. Prior studies indicate that increased chronic stress in depression could lead to limited cognitive processing, perhaps regardless of estrogen levels and exposure.^42^ The alteration of the hypothalamo-pituitary axis (HPA) hormones commonly seen in depression could lead to alterations in brain structure and function, such as decreased subgenual PFC and hippocampal volumes and an increase in amygdala volume.^43^ Estrogen hormone levels may modulate the stress system functioning in women through increasing neuroplasticity and maintaining active synapses in the prefrontal cortex and hippocampus.^13,42^ Estrogen treatment protects hippocampal synapses from the detrimental effects of acute cortisol increases; however, little is known about estrogen’s effects on chronic cortisol exposure often seen in depressive states.^13,42^

Limitations of this study include using a relatively small convenience sample. We did not correct for multiple comparisons in the analyses because of the exploratory nature of this project. In addition, there could have been retrospective recall bias for age of menarche, menopause, or hormone replacement therapy, although we have no reason to suspect that recall was biased in our sample. Additionally, the possible effect of surgical menopause on cognition and prior history of depression was not included in this secondary analysis. There was a significant age difference of approximately 3 years between the non-depressed and depressed groups of women, although we controlled for age in all our analyses. Finally, the generalizability and applicability of the present findings to older cohorts of women is limited, as our sample was a relatively younger old-adult population.

In conclusion, our findings suggest that CLEE can protect against memory decline in non-depressed older women. However, CLEE had no effect on cognitive function in the depressed group. Future studies need to evaluate the role of CLEE in brain aging, depression, and cognitive decline.

## Supplemental Material

Supplemental Material - The Effect of Cumulative Lifetime Estrogen Exposure on Cognition in Depressed Versus Non-Depressed Older Women08919887221090216 Supplemental Material for The Effect of Cumulative Lifetime Estrogen Exposure on Cognition in Depressed Versus Non-Depressed Older Women by Hanadi Ajam Oughli MD, Sarah A. Nguyen MD, Prabha Siddarth, PhD, Molly Fox, PhD, Michaela Milillo BS, Linda Ercoli, PhD, and Helen Lavretsky, MD, MS in Journal of Geriatric Psychiatry and Neurology
