# Supplementary material for: The Effect of Cumulative Lifetime Estrogen Exposure on Cognition in Depressed Versus Non-Depressed Older Women
Source: J Geriatr Psychiatry Neurol. 2022 Apr 11;35(6):832–9. doi: 10.1177/08919887221090216 (PMC9574476; doi:10.1177/08919887221090216)
Supplement: Supplemental Material - The Effect of Cumulative Lifetime Estrogen Exposure on Cognition in Depressed Versus Non-Depressed Older Women08919887221090216 [file sj-pdf-1-jgp-10.1177_08919887221090216.pdf]

**Supplementary Material: Reproductive Questionnaire**

"Hello ____. My name is _________. I'm calling from the UCLA Yoga and Memory Training Study.

You are receiving this call since you have either completed the Yoga and Memory Training study, or have participated in some parts of the study. I am reaching out regarding a new addition of questions to our demographics inventory, and we are trying to collect this information from as many participants as we are able to. These questions will be very useful for an anthropological analysis that we are doing with this study!

We understand the nature of these questions is very personal. If you feel uncomfortable and do not wish to answer these questions, please let me know if you would like to refuse to answer.

**New Questions:**

1) Number of siblings and birth order (For example: 2 sisters, I'm the second/middle child): ____________________________________________________________

2) Town/City/State/Country of Birth (as specific as possible!): ____________________________________________

3) Age at Menarche (first menstrual period): ____________

4) Number of complete pregnancies with live births: ___________

5) Number of complete pregnancies with live births followed by child being placed for adoption: _______________

6) Number of adopted children: __________

7) For each child, were you the primary caregiver: __________________________

   a.) Are your children still living: _______

   b.) If no, how old were they when they passed away: __________

8) Number of complete pregnancies with stillbirths: ___________

9) Number of incomplete pregnancies (miscarriages and abortions): ___________

For each child, did you breast feed and for approximately how long?:

Age at menopause: ___________

Duration of hormone replacement therapy use and type (Estrogen only, Estrogen + Progesterone, Estrogen + Progesterone + Testosterone): _____________

Thanks so much for your time today and for your participation. Good bye."
